# Robust automatic hexahedral cartilage meshing framework enables population-based computational studies of the knee

**DOI:** 10.3389/fbioe.2022.1059003

**Published:** 2022-12-09

**Authors:** Kalin D. Gibbons, Vahid Malbouby, Oliver Alvarez, Clare K. Fitzpatrick

**Affiliations:** Computational Biosciences Laboratory, Mechanical and Biomedical Engineering, Boise State University, Boise, ID, United States

**Keywords:** osteoarthritis, modeling, mesh generation, biomechanics, knee, finite element

## Abstract

Osteoarthritis of the knee is increasingly prevalent as our population ages, representing an increasing financial burden, and severely impacting quality of life. The invasiveness of *in vivo* procedures and the high cost of cadaveric studies has left computational tools uniquely suited to study knee biomechanics. Developments in deep learning have great potential for efficiently generating large-scale datasets to enable researchers to perform *population-sized* investigations, but the time and effort associated with producing robust hexahedral meshes has been a limiting factor in expanding finite element studies to encompass a population. Here we developed a fully automated pipeline capable of taking magnetic resonance knee images and producing a working finite element simulation. We trained an encoder-decoder convolutional neural network to perform semantic image segmentation on the Imorphics dataset provided through the Osteoarthritis Initiative. The Imorphics dataset contained 176 image sequences with varying levels of cartilage degradation. Starting from an open-source swept-extrusion meshing algorithm, we further developed this algorithm until it could produce high quality meshes for every sequence and we applied a template-mapping procedure to automatically place soft-tissue attachment points. The meshing algorithm produced simulation-ready meshes for all 176 sequences, regardless of the use of provided (manually reconstructed) or predicted (automatically generated) segmentation labels. The average time to mesh all bones and cartilage tissues was less than 2 min per knee on an AMD Ryzen 5600X processor, using a parallel pool of three workers for bone meshing, followed by a pool of four workers meshing the four cartilage tissues. Of the 176 sequences with provided segmentation labels, 86% of the resulting meshes completed a simulated flexion-extension activity. We used a reserved testing dataset of 28 sequences unseen during network training to produce simulations derived from predicted labels. We compared tibiofemoral contact mechanics between manual and automated reconstructions for the 24 pairs of successful finite element simulations from this set, resulting in mean root-mean-squared differences under 20% of their respective min-max norms. In combination with further advancements in deep learning, this framework represents a feasible pipeline to produce *population* sized finite element studies of the natural knee from subject-specific models.

## 1 Introduction

Osteoarthritis (OA) of the knee is increasingly prevalent as our population ages, affecting an estimated 654.1 million individuals aged 40 and over in 2020 worldwide, including 15.8% of the North American population (Cui et al., 2020). Patients suffering from OA report joint pain and stiffness, cracking or grinding noises with joint movement, and decreased function and mobility. These symptoms and prevalence has made OA a leading cause of pain and disability worldwide, representing a significant economic burden of approximately 2% of a given country’s global domestic product (O’Neill et al., 2018). The disease is characterized by a deterioration of the cartilage, tendons and ligaments, and the development of osteophytic bone spurs within the joint (Lane et al., 2011). The study of knee OA presents several challenges to researchers, it is a multifactorial joint disease—confounding subject-specific factors include geometry, biomechanics, biology, and mechanobiological adaptations (Dell’Isola et al., 2016; Paz et al., 2021) — making it difficult to isolate features driving disease progression.

Researchers are limited in their ability to collect *in vivo* biomechanical data relating to the knee, with some researchers relying on externally attached pressure transducers, motion capture, electromyography (Paz et al., 2021), or using implants with telemetric sensors following joint replacement to estimate joint forces (Wang et al., 2015; Almouahed et al., 2017). *In vitro* studies relying on cadaveric tissue and joint specimens using mechanical joint simulators have been conducted in the past (DesJardins et al., 2000; Maletsky and Hillberry, 2005; Varadarajan et al., 2009; Colwell et al., 2011). Financial barriers associated with sourcing cadaveric specimens and employing surgeons to perform surgeries or joint assessments can be prohibitively high, limiting the scope of most cadaveric studies to a small number of subjects or activities. With their relative cost-effectiveness and inherent non-invasiveness, computational studies aim to complement *in vivo* and *in vitro* studies, using material properties and joint mechanics data from these studies to validate computational analyses.

Researchers can use validated models to simulate activities of daily living (Torry et al., 2011; Ivester et al., 2015) and, with large-volume simulations, they can use these data to link geometric and kinematic features to force and contact mechanics outputs using classic methods of inferential statistics (Bryan et al., 2010; Fitzpatrick and Rullkoetter, 2012; Gibbons et al., 2019). These statistical models are simple to use and require orders of magnitude less computing time when compared to the simulations they are derived from, making them more suitable for clinical applications. However, given the variability inherently present across the population, studies require hundreds or even thousands of subjects to adequately capture the spectrum of variability present across the population and develop reliable statistical models based on these data. Unfortunately, several bottlenecks have limited researchers to using simple parameterized or synthetically generated joint geometries in the past.

Developing a working FE simulation of a single knee typically begins with medical images, which then undergo segmentation, reconstruction, meshing, and mesh registration. Traditionally, the primary bottlenecks preventing clinical adoption are segmentation and meshing, which may take several days work per knee (Bolcos et al., 2018; Cooper et al., 2019). Recent advances in deep learning are reducing the segmentation process from hours or days of person-hours to only minutes of computing time, but are limited by the availability of training data (Ambellan et al., 2019; Burton et al., 2020; Ebrahimkhani et al., 2020). In FE simulations, hexahedral meshes are optimal for contact regions (e.g., articular contact of cartilage surfaces), as tetrahedral meshes overestimate stiffness while requiring a larger number of elements (Ramos and Simões, 2006; Tadepalli et al., 2011). While automatic triangular surface and tetrahedral volume meshing algorithms have existed for decades, robust hexahedral meshing algorithms are still being actively researched (Ito et al., 2009; Gregson et al., 2011; Livesu et al., 2020, 2013; Guan et al., 2020). Past researchers have used templated hexahedral meshes with control nodes to create fitted approximations of subject geometries (Baldwin et al., 2010; Rao et al., 2013), or custom swept-extrusion meshing algorithms validated on a small number of subject geometries (Rodriguez-Vila et al., 2017). However, to the authors’ knowledge, no researchers have successfully generated subject-specific hexahedral knee cartilage meshes for hundreds of subjects in a fully automated fashion. The present study aims to answer the questions: 1) can automatic segmentation and meshing algorithms allow us to generate hundreds of *patient-specific* simulations in a fully automated fashion; 2) how closely do finite element meshes derived from deep learning segmentation labels match their manually segmented counterparts; and 3) how sensitive are the final simulation results to the predicted tissue labels?

## 2 Methods

**FIGURE 1:**
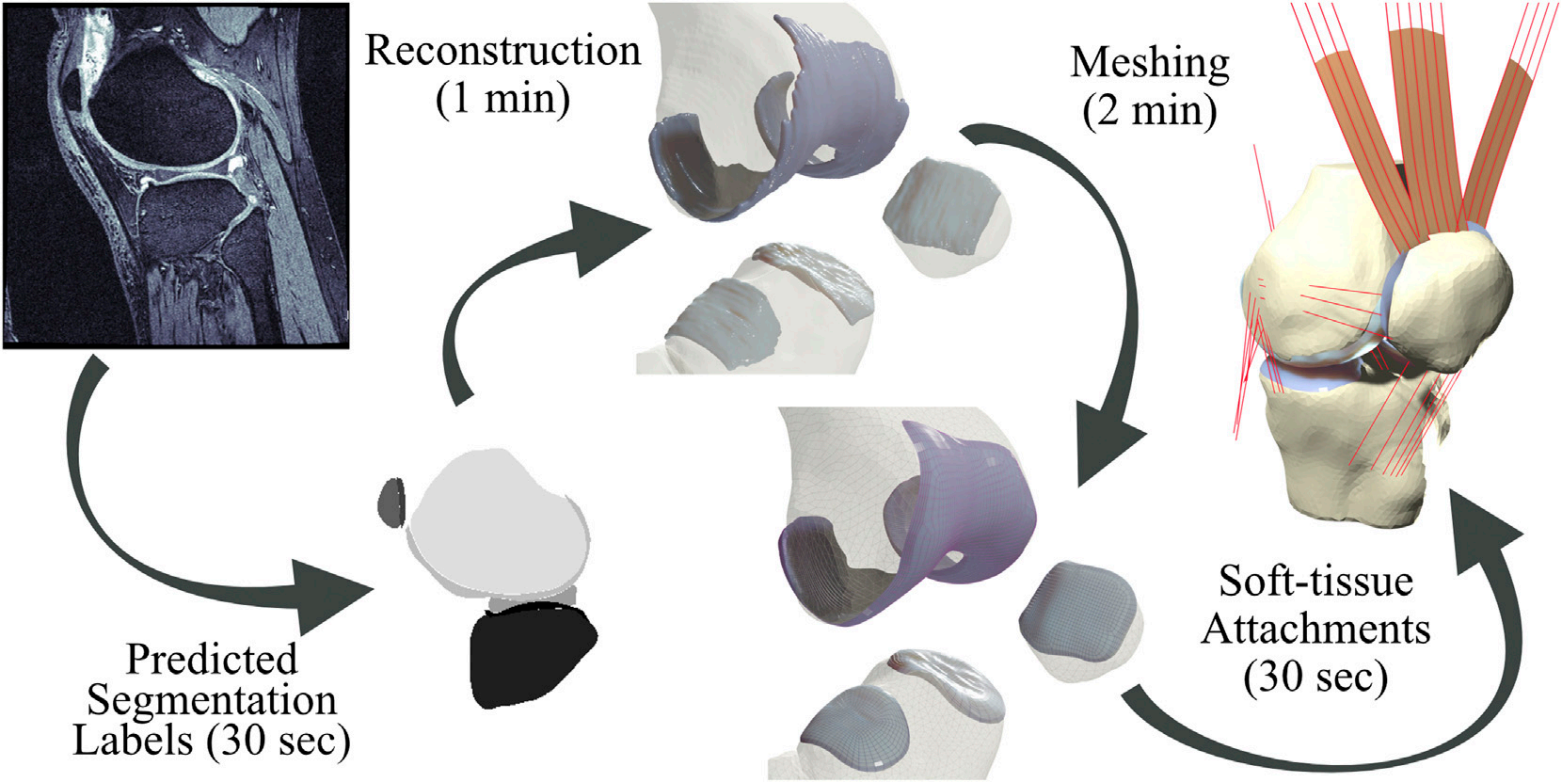
Pipeline from magnetic resonance images to simulation-ready finite element meshes. Segmentation labels produced by a neural network are used to produce reconstruction geometries. Bone geometries are then meshed using first-order triangular surface elements, and cartilage tissues are meshed using a customized swept-extrusion hexahedral meshing software. Soft-tissue attachment sites are placed using a nearest-neighbor search with a registered template mesh, resulting in a turn-key mesh ready to drop into existing finite element simulations.

To answer these questions, we have implemented a completely automated imaging-to-simulation pipeline (Figure 1). We used a simple convolutional neural network (CNN) to automate segmentation and we applied established visualization toolkits for geometric reconstruction and registration (Schroeder et al., 2006; Zhou et al., 2018; Sullivan and Kaszynski, 2019). We used standard triangular mesh generation tools to perform rigid body bone meshing (Hoppe et al., 1993; Valette et al., 2008). For the hexahedral cartilage meshes, we ported a publicly available Matlab-based hexahedral swept-extrusion algorithm (Rodriguez-Vila et al., 2017) to Python, and then customized and expanded upon this algorithm until it was sufficiently robust to produce hundreds of meshes. We subsequently enhanced the algorithm with a custom cartilage-to-bone interface blending algorithm and soft-tissue attachment site locator using a templated mesh with nearest neighbor search. This resulted in an efficient pipeline from image sequence to FE-ready mesh. We ran the output meshes in a simulated 90° knee flexion activity in three batches: our entire *manual* data set (train, validation, and test) excluding the added blending algorithm, again with blending, and the reserved test dataset utilizing *predicted* segmentation labels.

**FIGURE 2:**
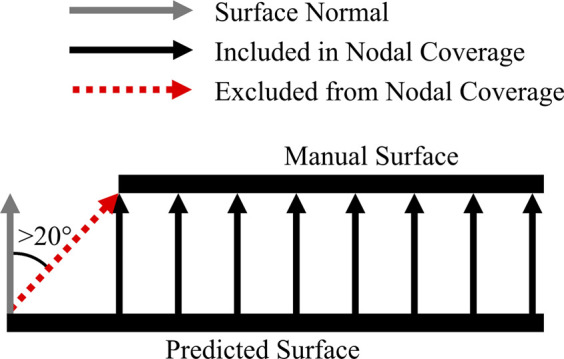
Exclusion criteria for nodal coverage. If the rays directed from the predicted surface to the nearest point on the manual surface deviated from the predicted surface normal by more than 20° , that ray was excluded from nodal normal calculations.

To quantify the effect of predicted segmentation labels on resulting meshes, we compared articular surface deviations between manual and predicted segmentations. We traced rays from the nodes of the predicted mesh to the nearest surface of the manual mesh and used these data to compute distributions of distances. To account for mismatches in overlapping edges, we only included rays within 20° of the surface normal which we reported as *percent nodal coverage* (Figure 2). Further down the pipeline, we ran FE simulations to predict contact pressure and area joint mechanics for the medial and lateral sides of the tibial cartilage tissues, and then compared these metrics between manually and automatically generated datasets using root-mean-square (RMS) differences.

### 2.1 Data source

We sourced image sequences and their respective segmentation labels for the knees of 88 subjects from the Imorphics dataset (Paproki et al., 2014), which is part of the publicly available Osteoarthritis Initiative database (National Institute of Arthritis and Musculoskeletal and Skin Diseases (NIAMS, 2004). Each subject attended baseline and 12-month follow up appointments, resulting in 176 image sequences, containing 3D double echo steady-state images consisting of (384 × 384 × 160) voxels with a spatial resolution of (0.37 × 0.37 × 0.70) mm in the sagittal plane. Segmented tissues included the menisci, femoral, patellar, and tibia cartilage with independent medio-lateral labels for the menisci and tibial cartilage. As stated in Paproki et al. (2014), one person, who trained under both an expert in segmentation and a musculoskeletal radiologist, performed the manual segmentations. Additionally, this user achieved an intra-observer coefficient of variation less than 3% on paired test images within the Imorphics cartilage segmentation training protocol, with the expert reviewing their final segmentation maps.

### 2.2 Image segmentation

We used a textbook 2D encoder-decoder CNN based on the popular U-Net architecture (Ronneberger et al., 2015) to perform automatic image segmentation. Each contractive block made use of batch normalization and rectified linear unit activations, while using strided convolution layers to downsample the image feature maps. We implemented residual connections around each contractive block. The network was four contractive-expansive blocks deep, with a final softmax activation. We augmented input images with up to a 30° rotation 50% of the time, as well as randomized brightness and contrast, elastic transformations, and grid distortions a maximum of 30% of the time. We trained this network on the image-label observations while reserving 14 *subjects* (each subject at baseline and 12-month timepoints, totaling 28 sequences) for a validation dataset used to detect overfitting, and reserved an additional 14 *subjects* (again, at both timepoints) for a final test set, unseen during training. We trained the model until the mean Dice similarity coefficient (DSC) of the validation set reached 89% (Taha and Hanbury, 2015). Resulting labels were based on probability scores for the four articular cartilage tissues present in the Imorphics segmentations, with our three additional bone tissues.

### 2.3 Reconstruction of tissue geometry

Segmentation labels for each tissue, whether processed manually or predicted, then underwent morphological closing with a five-voxel (cartilage) or three-voxel (bone) uniform kernel to remove segmentation artifacts before we reconstructed the surface using marching cubes (Lorensen and Cline, 1987). After viewing a subset of these raw surface reconstructions, we assumed all cartilage tissues consisted of singular connected volumes. We treated all but the largest enclosed volumes as segmentation artifacts, which we discarded. We then applied a decimation filter resulting in an 80% reduction in surface triangle density. The final step included nine iterations of Laplacian smoothing for all tissues. For every *bone* geometry, we applied the MeshFix algorithm to correct triangle intersections, singularities, or degenerate elements (Attene, 2010). To preserve physiological holes within *cartilage* tissues, we limited MeshFix to reversing inward-facing normals.

### 2.4 Meshing

For the tibia, femur, and patella bones, we created uniform triangular rigid body surface meshes using Voronoi clustering (Valette et al., 2008), with a target element size of 3 mm. For the remaining tissues, we ported an existing open-source cartilage meshing algorithm (Rodriguez-Vila et al., 2017) to the python programming language. The algorithm used a swept set of point origins and raytracing to place two matching rectangular grids along the bone-side and joint-side surfaces of cartilage reconstructions. We then connected these matching grids to form an initial set of ill-conditioned hexahedral elements, with a portion of the elements on the cartilage edges containing six nodes. We repaired these degenerate elements through the creation, deletion, or merging of nodes and edges. Once fully connected, the mesh underwent optimization to ensure every element had a non-negative scaled Jacobian (SJ). Individual elements then underwent an in-plane—not depth wise—subdivision to become four elements before undergoing an iterative optimization and smoothing process until every element’s SJ was above 0.5. Finally, we subdivided the cartilage depth into multiple elements. Our parameter choices resulted in average element edge-length of approximately 1 mm, with cartilage depths divided into four linearly spaced elements.

#### 2.4.1 Hexahedral meshing algorithm modifications

Excluding a cartilage mesh blending step (detailed in Section 2.4.2 below), the bulk of our changes consisted of replacing low-level mathematical calculations with functionality built into 3D *Python* libraries, encapsulating novel logic into functions, and developing an object-oriented application point interface with unified helper methods allowing for simplified plotting and cell quality calculations during any step following creation of the ill-conditioned mesh. Making these changes allowed us to improve vectorization, locate and fix typographical errors resulting from repeated logic, and will facilitate future algorithm adaptation for other joints. We added patellar cartilage meshing by adapting the femoral cartilage algorithm. The initial tibial cartilage meshing algorithm based on raytracing an interior grid with radial sectors was prone to failure. Discarding back faces during a planar projection and basing placement of the interior grid on a *scaled* bounding box improved robustness. Meshing can still fail if a cartilage hole lies on the edge of the interior grid, so we added a fallback method using a rectilinear grid of rays bounded by the *unscaled* bounding box.

A primary failure mode of the original package was looping infinitely during the final mesh optimization, which we traced back to the misclassification of degenerate elements. We included additional controls to more accurately classify the configuration of six-node elements (*peaks*, *mirrors*, three element *stairs*, *steps* consisting of two elements, and single element internal *corners*), with each configuration treated separately to eliminate negative volumes or extreme skewed elements that resulted in an oscillating optimization solution. For example, we implemented logic confirming degenerate nodes were at the same topological indices when detecting step degenerates, which previously only checked if neighboring elements shared a single edge.

#### 2.4.2 Cartilage mesh blending

**FIGURE 3:**
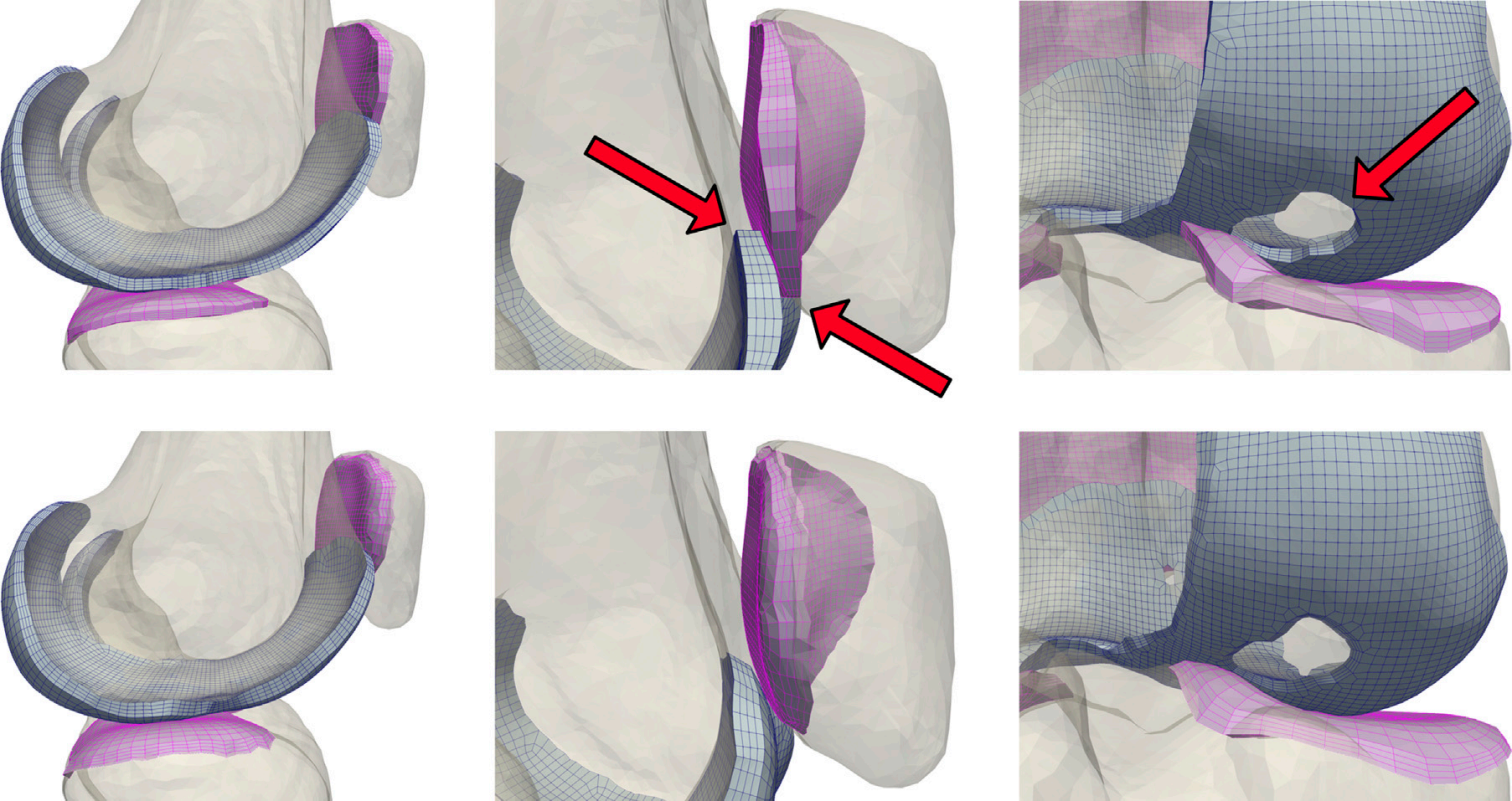
A comparison of unblended (top) and blended (bottom) cartilage profiles. Dynamic activities involving tangential motion of cartilage interfaces can result in the edges of articulating meshes catching on each other, causing simulation failures. This occurs most frequently near the trochlear groove of the patellofemoral joint, and in knees with significant cartilage loss in the tibiofemoral joint.

Modeling cartilage as linearly subdivided brick elements results in a step interface with bones (Figure 3). These sharp corners can cause unresolvable impingement issues and unrealistic edge loading when modeling joint contact. As articulating surfaces transition from cartilage-bone to cartilage-cartilage contact, there is risk of simulation failure due to protruding nodes on the cartilage edge unable to resolve the excessive nodal forces and geometric constraints generated through contact between the corners of articulating meshes. Issues intensify when modeling subjects with significant cartilage degradation, as they frequently exhibit total cartilage loss near the trochlear groove of the patellofemoral joint, or holes within the tibiofemoral joint. To rectify this, we developed and incorporated a mesh blending step before the final depth-subdivision.

**FIGURE 4:**
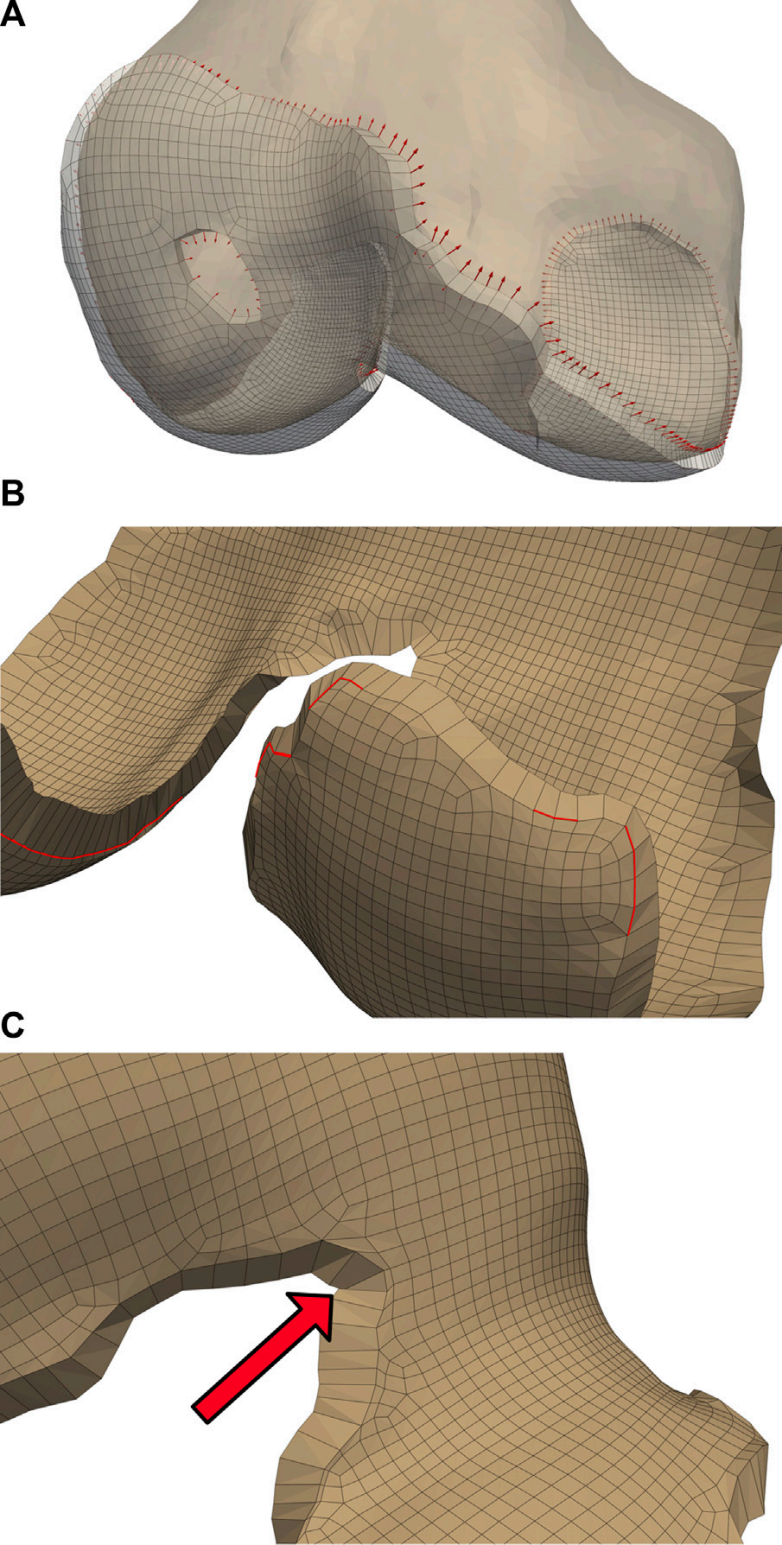
Initialized displacements for cartilage mesh blending. Vector lengths are based on cartilage depth at the edge, with 80% of the depth assigned to the bone-side nodes and the remaining 20% assigned to joint-side nodes **(A)**. Feature angles of the blended edge are required to be greater than 35° . The nodes attached to the red lines will have their displacement relaxed in 10% increments **(B)**. Element intersections caused by blending displacement, which occur frequently within cartilage holes and internal corners of the femoral cartilage. Savitsky-Golay filtering of the edge nodal location components can solve a case such as this **(C)**.

The blending algorithm operated on quadrilateral elements extracted from the *single-layer* hexahedral mesh surface. Smoothing the cartilage-bone transition required stretching the edge nodes of the *bone-side* cartilage surface towards the exterior while compressing the corresponding nodes of the *joint-side* surface. We calculated the direction of these displacements using point normals of the mesh faces of the cartilage edges, then displaced the nodes until they created a 45° slope (Figure 4A). We weighted the bone-side nodes to perform 80% of the displacement. If possible, we attempted to translate the bone-side nodes to a paired bone mesh using a nearest element surface search between the bone elements and bone-side nodes of the cartilage mesh.

Some elements became skewed after displacement, which we corrected by iteratively reducing the bone-side nodal displacements until all joint-side edges attained a minimum feature angle of 35° (Figure 4B). We selected this feature angle through assessing a range of angles, and determining that this value resulted in a reasonable trade-off between sufficiently smooth interfaces, correcting for element skewness, and minimizing changes in cartilage surface area and computing time. Element intersections may appear along the edges of interior curves and small holes within the cartilage interior (Figure 4C), which we attempted to correct by smoothing the nodal locations of the edges with a 3rd degree Savitzky-Golay filter (Savitzky and Golay, 1964). If element intersections remained, we iteratively reduced the magnitude of our nodal displacements by 10%.

**FIGURE 5:**
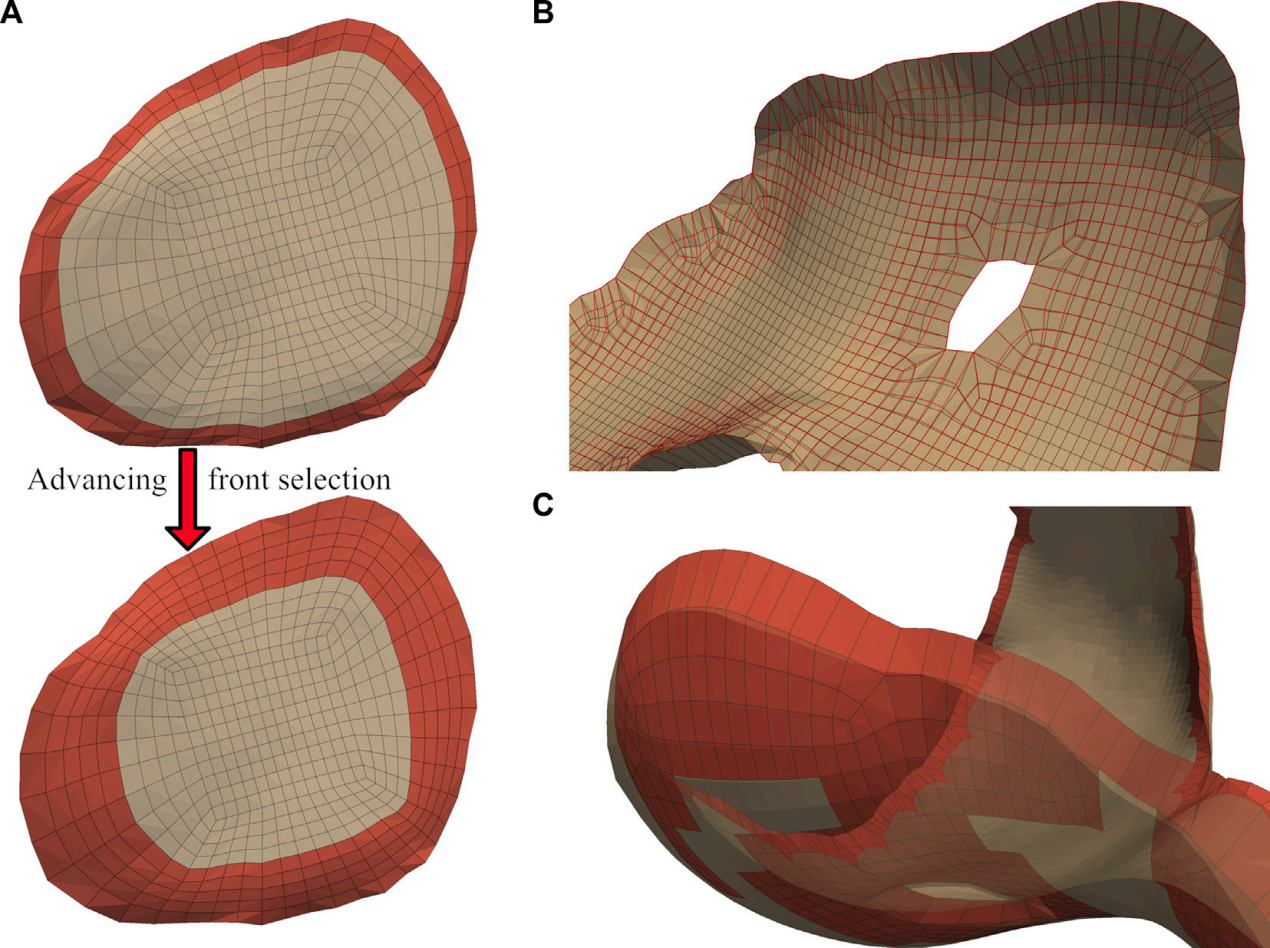
A graduated smoothing is performed using an advancing front of surface face selections **(A)**, independently for the joint- and bone- -side surface faces. The nodes on the cartilage edges are constrained while Laplacian smoothing iterations are reduced each time the front advances. The bone-side surface feature angles are now reduced to 30° **(B)**, while the joint side smoothing front advances once, but with a higher number of initial smoothing iterations **(C)**.

We applied independent Laplacian smoothing operations to the bone- and joint-side surfaces. We performed each smoothing operation iteratively, using an advancing front of quadrilateral faces beginning at the bone-side edge (Figure 5A). For each advance in the selection front, we decreased the number of smoothing iterations, resulting in a decrease of nodal displacements within the interior of the cartilage mesh. For the joint-side surface we stopped before elements skew, or when the blended feature angles reached a minimum of 30° (Figure 5C). For the bone-side surface, the front progressed from an element depth of two until five (Figure 5B), with the number of smoothing iterations halved for each advance. We empirically selected the initial number of smoothing iterations to be $\frac{550}{n_{surface}}\;\left(n_{surface}-n_{front}\right)$ , where $n_{surface}$ represented the number of quadrilateral faces present in the cartilage, and $n_{front}$ was the selection subset. We required that interior elements had SJs exceeding 0.5 and blended element SJs remained positive.

#### 2.4.3 Soft-tissue attachment locator

We mapped soft-tissue attachment sites from a manually segmented knee geometry based on MRI imaging with 160 manually segmented soft tissue attachment sites. We used this *template* mesh for the automatic selection of attachment sites for the Imorphics meshes. We registered input femoral meshes to the template using an iterative closest points algorithm (Chen and Medioni, 1992), with the resulting transformation applied to the remaining tissues. We applied a nearest neighbor search for each soft-tissue attachment site and nodal coordinates defining joint axes, before applying an inverse-transformation back to the original scaling and position (Ta, 2019; Malbouby et al., 2022).

### 2.5 Knee flexion simulation

We adapted the knee flexion simulation from a previously published model of the implanted knee (Fitzpatrick and Rullkoetter, 2014, 2012; Gibbons et al., 2019), using the commercial FE solver, Abaqus/Explicit (Dassault Systèmes). Briefly, we applied knee loads and muscle forces through mechanical actuators, which we implemented using force- or moment-driven connector elements. We adopted ligament soft-tissue properties from a previously published study where passive laxity tests performed on a series of four cadaveric knees were used to calibrate reference strain and linear stiffness values of the major tibiofemoral ligaments (Harris et al., 2016). To create a 6-degree-of-freedom joint, we applied anterior-posterior force and internal-external torque to the femur, with medial-lateral translation free. We simulated knee flexion by balancing a vertical load applied at the hip with quadriceps and hamstring loads controlled by a proportional-integral controller implemented through a user subroutine. We derived flexion and joint loading profiles from data reported from five patients with telemetric knee implants (Heinlein et al., 2007; Kutzner et al., 2010). Due to the large number of simulations required, we excluded material deformation from cartilage representations–instead using linear pressure-overclosure contact definitions to compensate for rigid cartilage elements within the patellofemoral and tibiofemoral joint complexes (Halloran et al., 2005; Fitzpatrick et al., 2010; Hume et al., 2020).

## 3 Results

**FIGURE 6:**
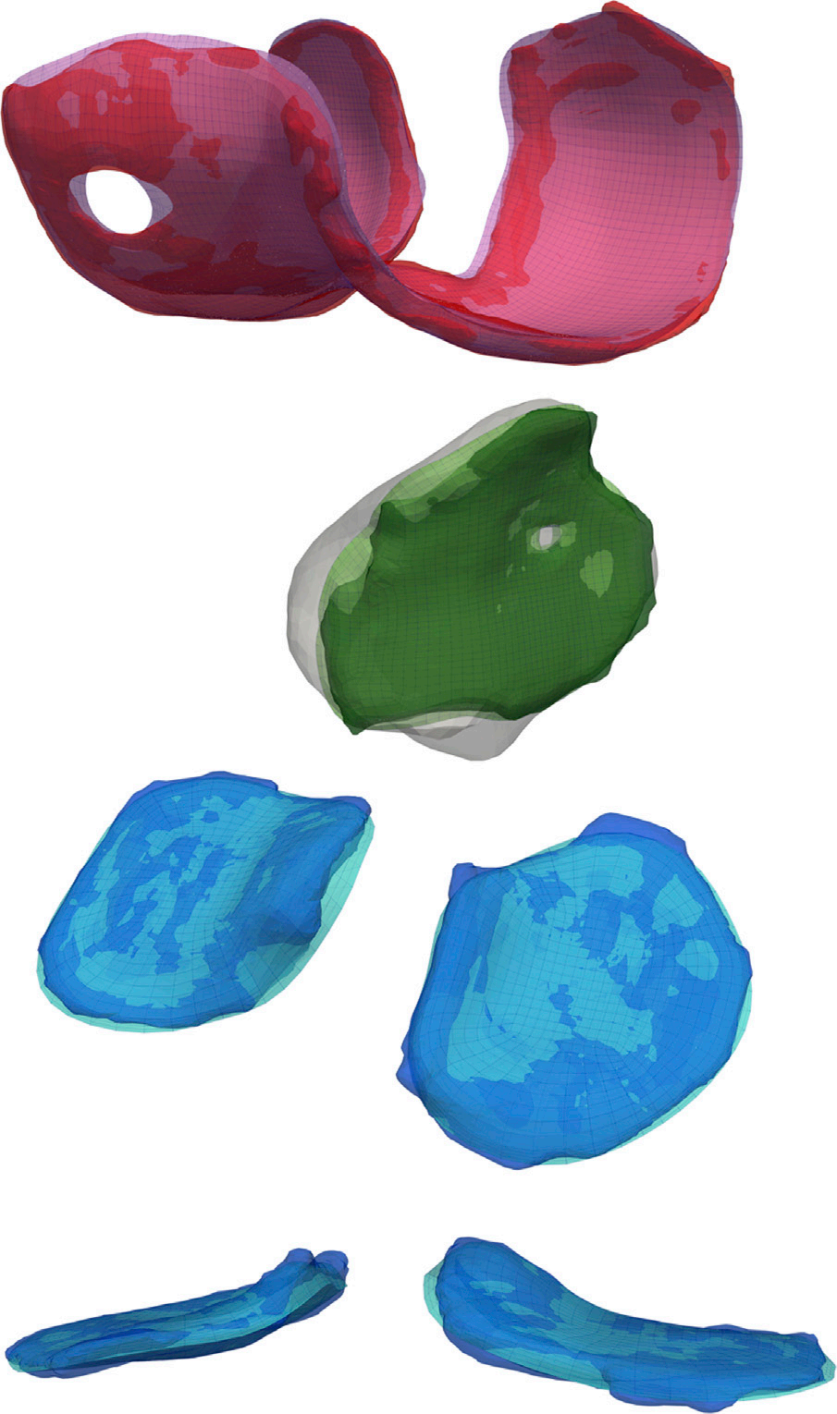
A comparison of the reconstructed geometry with triangular surface bone and hexahedral volume cartilage meshes. Blending the edges of the cartilage mesh will constrict holes and increase the planar surface, but the authors believe these negatives are offset by the reduction in joint dislocations during simulated activities.

We used the original algorithm to analyze a subset of 23 knee reconstructions from the Imorphics dataset, with only six successfully meshing. After porting, implementing bugfixes, and adding additional degenerate element detection cases, our meshing algorithms successfully produced “watertight” bone and cartilage meshes with high-quality elements for all 176 image sequences, for both the manual and predicted segmentation maps (Figure 6). The average time to mesh eight tissues with cartilage blending was one min and 22 sec, with a maximum time of four min and nine sec. We performed these computations on an AMD Ryzen 5600X processor, using a parallel pool of three workers for bone meshing, followed by a pool of four workers meshing the four cartilage tissues.

Of the 704 total cartilage tissues that we meshed using manual segmentations, 87.4% resulted in blended meshes with every bone-side node fused to the bone surface. Of the 86 tissue meshes that failed to fuse the entire bone-side surface, 10 more were able to fuse using only the perimeter edge. The remaining 10.8% of all attempted geometries were blended without reference to the underlying bones. For the blended tissues, the five edge layers of elements altered by the algorithm saw average reductions in SJ qualities between 5.23% and 46.1%. The edge most layer saw the biggest decrease, from a mean SJ of 0.86 to 0.46. The remaining layers saw less than 25% reduction. Of the 352 tibial cartilage geometries, we needed to mesh 17 using a simplified grid (Table 1). Running each manually segmented simulation without cartilage blending resulted in 76.9% completing the flexion activity. Adding blending increased our success rate to 89%.

**TABLE 1:** Distribution of cartilage meshing fallback algorithms. If issues occur during the meshing process, features are turned off beginning with fusing bone-side cartilage nodes to the nearest bone surface, followed by a reduction in the nodal displacements during cartilage-to-bone blending. For the tibial cartilage, a simplified rectilinear grid can be used during raytracing, instead of a more complicated distribution based on sectors. Finally, a planar subdivision step may be performed earlier in the algorithm to better capture complicated cartilage edges, at the expense of speed.

	Count	Total	Percent (%)
Cartilage meshed successfully without fallbacks	615	704	87.4
Tibia cartilage meshed with simplified grid	17	352	5.40
Grid-based meshing required in-plane subdivision before raytracing	15	371	4.04
Interior bone-side surface nodes failed to fuse to bone surface	86	704	12.2
All bone-side nodes failed to fuse to bone surface	76	704	10.8

**FIGURE 7:**
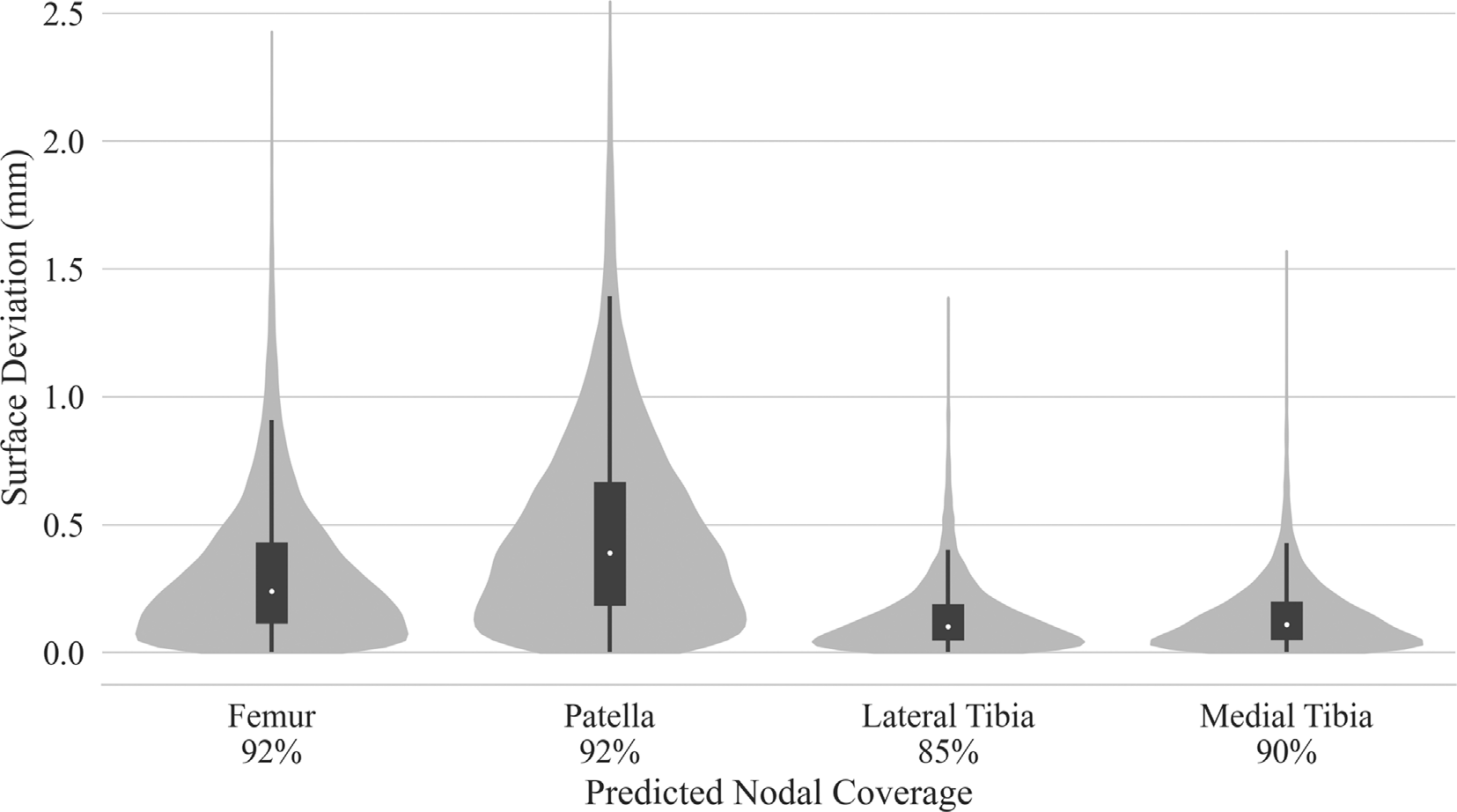
Kernel density estimated distributions of distance between predicted and manually segmented mesh surfaces, with box plots superimposed. Distances were traced from the nodes of the predicted mesh until intersection with element faces of the manual mesh. Nodal coverage denotes sample size determined by traced rays deviating no more than 20° from node normals at the base of each ray.

**FIGURE 8:**
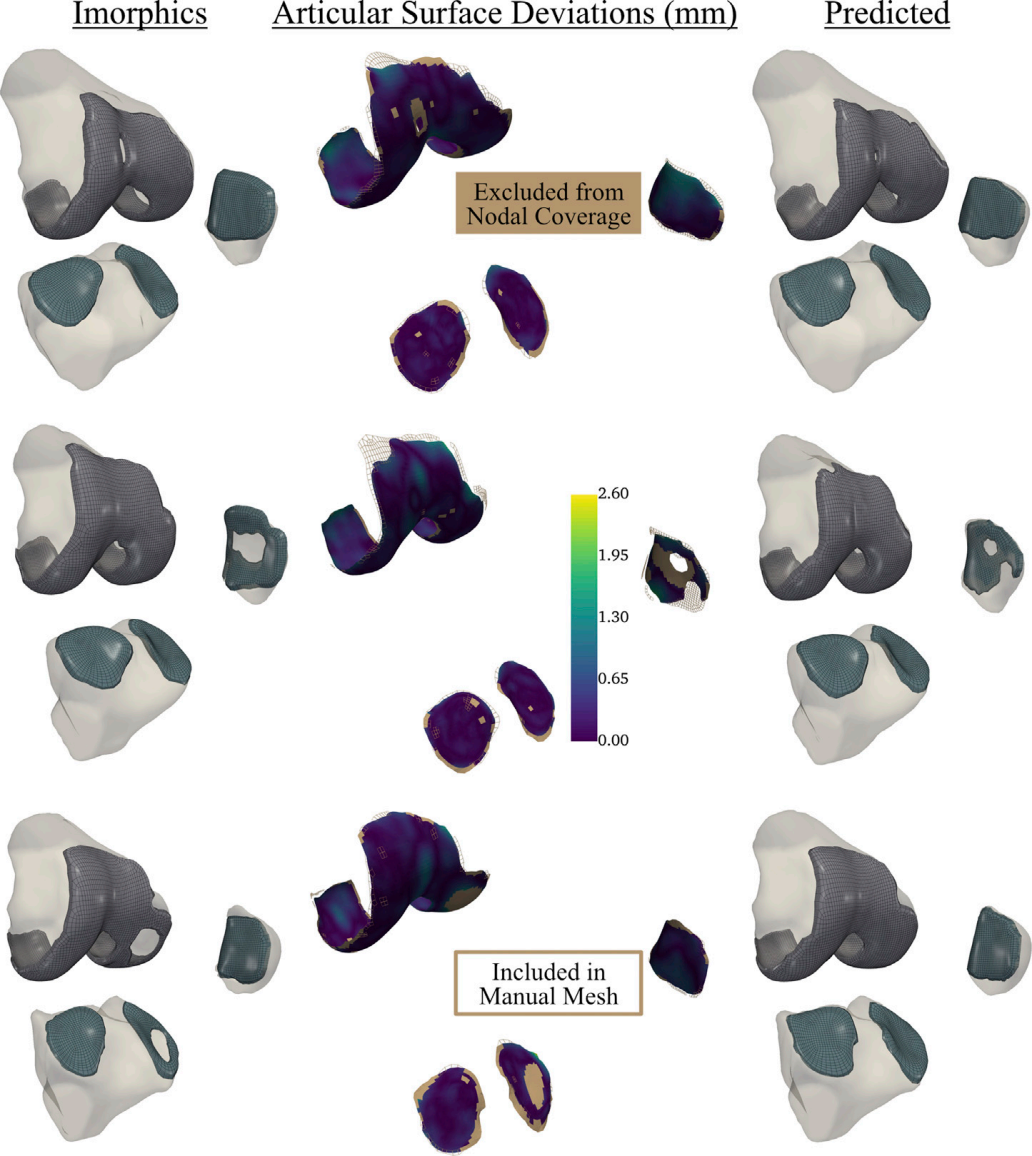
Articular surface deviations comparing representative meshes derived from the neural-network predicted segmentation labels, and the provided Imorphics labels from a reserved test dataset. Nodal coverage is represented by the scalar bar, while nodes within the solid tan region were excluded from distance calculations. This network tended to constrict cartilage holes, and would need fine-tuning before being used to study osteoarthritis. However, this also showcases the robustness of our automatic hexahedral meshing program, as each knee contained holes, of varying sizes, in one or more tissues.

Test dataset DSC scores for the bones were each above 97%. Cartilage DSC scores for the patellar and lateral tibial cartilage were 79% and 77%, while the femoral and medial tibial cartilage resulted in scores of 84% each. Articular surface overlap and conformity between the predicted and manually derived cartilage meshes are shown in Figure 7; Figure 8. Nodal coverage of the predicted meshes was between 90% and 92% except for the lateral tibial cartilage, which had coverage of 85%. Kernel density estimates resulted in right-skewed distributions, with the patellar cartilage resulting in the highest median deviation of 0.39 mm and 75% of patellar deviations below 0.46 mm. The remaining cartilage meshes had median surface deviations of 0.23 mm or less, and 75% of their deviations were less than 0.27 mm. Maximum outlier deviations fell between 1.39 mm and 2.54 mm.

**FIGURE 9:**
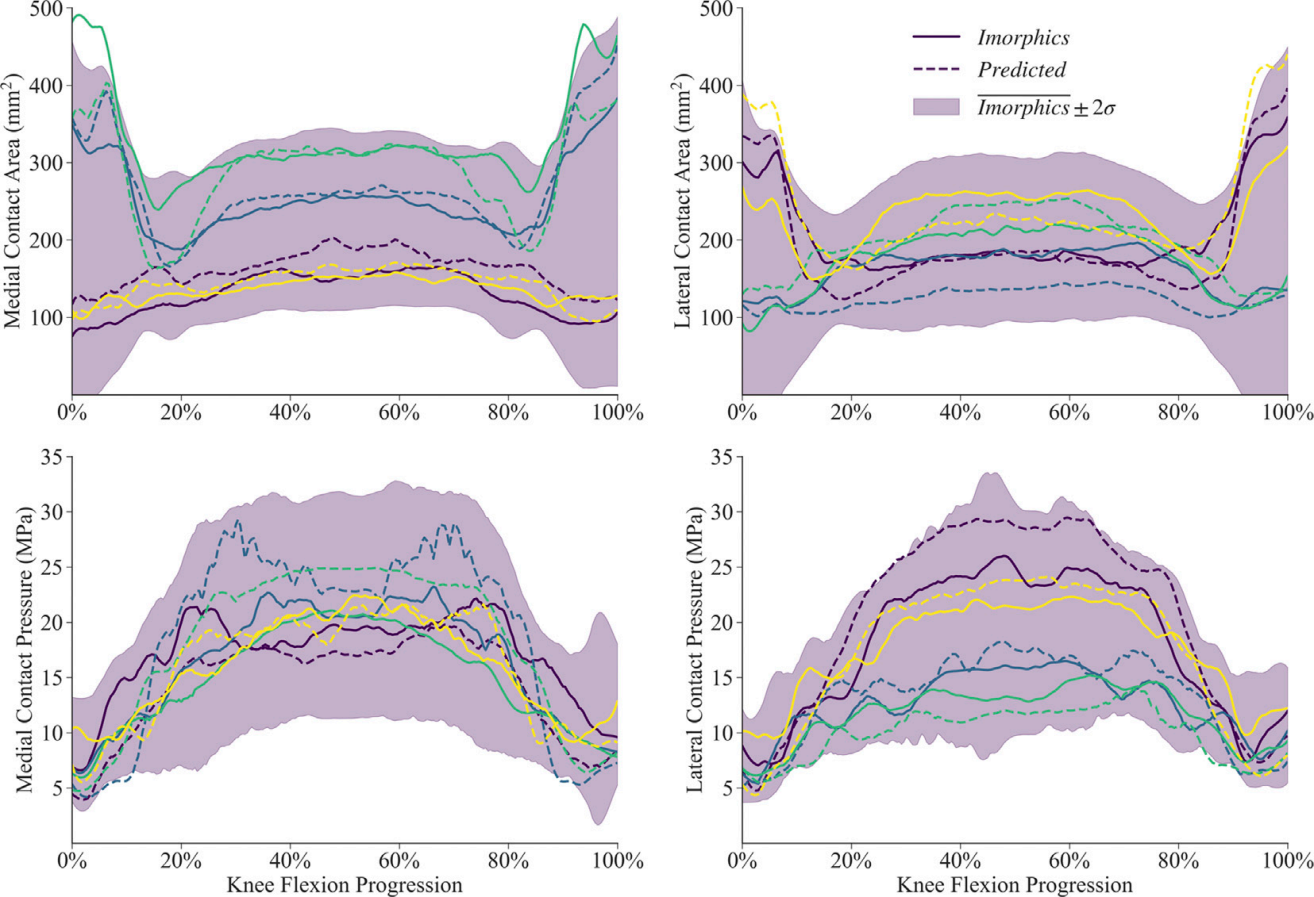
Contact mechanics data generated by 24 sets of segmentation labels provided with the Imorphics dataset and those predicted by a neural network. The predicted meshes tendsed to underestimate compressive pressure—and overestimate contact area—while the knee was nearly extended. Agreement throughout the middle 60% of the flexion cycle was excellent except for an overestimated medial contact pressure. Manual and predicted results for four representative simulations are shown for comparison.

Simulations for the manual and predicted test datasets both succeeded 92% of the time, with independent failures leaving 24 matched comparisons between manual and predicted FE simulations. Simulated contact mechanics for those remaining resulted in mean normalized RMS differences were below 20% for both medial and lateral sides, and remained under 22% for the 75th percentile (Table 2). The worst case resulted in a medial contact pressure error of nearly half the min-max range, more than doubling the 75th percentile value. For the initial and final 15% of the flexion activity, predicted meshes tended to underestimate compressive pressure while overestimating contact area (Figure 9). Agreement throughout the middle 60% of the flexion cycle was excellent except for an overestimated medial contact pressure.

**TABLE 2:** Contact area and pressure root-mean-squared error for tibial cartilage of the test dataset. Min-max normalized error terms are reported as percentages. Two of the predicted and an independent two from the manual sequences failed to finish the flexion activity, leaving 24 sequences for comparison.

	Contact area mean RMS error (mm^2^)		Contact pressure RMS error (MPa)	
	Medial	Lateral	Medial	Lateral
Mean	43.9 (15.7%)	25.1 (9.94%)	4.71 (19.50%)	3.21 (13.7%)
Std. Dev	23.3 (7.75%)	13.6 (4.33%)	2.15 (9.52%)	1.35 (4.38%)
Minimum	15.0 (5.04%)	8.16 (5.59%)	1.79 (8.55%)	1.67 (8.55%)
25%	25.4 (9.74%)	13.5 (6.92%)	3.40 (12.7%)	2.27 (11.3%)
50%	36.0 (13.6%)	25.6 (8.12%)	4.29 (18.2%)	2.70 (12.4%)
75%	63.1 (20.7%)	29.2 (11.9%)	5.65 (21.6%)	3.98 (15.0%)
Max	88.3 (29.5%)	62.9 (21.1%)	11.1 (48.3%)	7.07 (23.5%)

## 4 Discussion

Our first study objective was to develop automatic segmentation and meshing algorithms that would allow us to generate hundreds of working simulations in a fully automated fashion. We were able to create “watertight” triangular surface bone and high-quality hexahedral volume cartilage meshes for all 176 image sequences, both manual and predicted, in the Imorphics dataset. While 11% of our image sequences did not result in successful FE simulations, each of the failures exhibited excessive cartilage degradation resulting in bone contact which was not sufficiently captured in our current model definitions. This is a unique challenge when modeling osteoarthritic patients, as osteophytic bone spurs may increase the probability of deleterious bone-cartilage contact. However, a common mode of failure is a sharp cartilage edge contacting the bone, causing excessive deformation and stress concentrations at the cartilage edge elements—exactly what our blending algorithm aims to correct. Adding the blending algorithm allowed an additional 21 simulations to run successfully, while only affecting five edgewise layers of elements. For our simulation parameters, we saw a 46% reduction in SJs for the first 0.5 mm of cartilage edge, which decayed to between 5% and 25% for the next 4 mm from the edge. However, in order to achieve a success rate closer to 100%, we must perform additional analysis and iteration of bone-cartilage contact definitions to determine a set of contact and meshing parameters that are optimally compatible with prominent osteophytes commonly present in the OA population.

Our next objective was to quantify the effect of segmentation labels generated by a CNN on downstream FE meshes. Articular surface conformities with sub-millimeter median deviations and a minimum of 85% and 92% surface overlap for the tibiofemoral and patellofemoral joints, respectively, are likely improvements over statistical shape modeling or mesh templating for aggregate population studies, which lack the subject-specificity of models developed from patient-specific imaging. However, we may require further improvement in algorithm accuracy before these data become clinically useful. While there are more sophisticated algorithms available, we implemented a relatively simple CNN, but that same simplicity makes it an accessible choice for applied researchers outside of computer science, using consumer hardware. Our test dataset cartilage DSC scores ranging from 77% to 84% leaves room for improvement, but our meshing and soft-tissue attachment algorithms handled resulting geometric differences without issue. However, surface deviations were found to be driven by nodal overlap; our CNN tended to shrink or fill cartilage holes when compared to the manual reconstructions, which resulted in surfaces around mismatched holes pulling away from each other following the optimization and blending steps. Mitigating this effect using more sophisticated models, such as those proposed by Ambellan et al. (2019), Gatti and Maly (2021), and Tack et al. (2018), will be critical for studying damaged tissue. Some researchers have shown that CNNs trained on osteoarthritic datasets improve when tested on healthy tissue (Gatti and Maly, 2021), so the effect may be less pronounced if our model was applied to healthy knees.

Our final objective was to quantify how predicted segmentation labels affect FE simulation joint mechanics results. Our data shows that 75% of simulation contact pressure and area results deviate by less than 22%, and that most of the error occurs while the knee is extended. Our current CNN may not be suitable for studying contact mechanics during activities at lower flexion angles. Researchers should be mindful that errors introduced at the segmentation stage compound while traveling through the pipeline, altering soft-tissue attachments, for example.

While the primary focus of the current study was the development of an algorithm that could be used to generate robust finite element meshes for large-cohort populations, the finite element simulation that we have used to demonstrate the implementation of our algorithm is relatively simple, and as such, has a series of limitations and assumptions that should be noted. In order to run the hundreds of simulations required for this analysis in a computationally efficient manner, we did not allow for material deformation of the cartilage tissues, instead using linear pressure-overclosure definitions to compensate for rigid cartilage representations (Halloran et al., 2005; Fitzpatrick et al., 2010; Hume et al., 2020). The computational cost of these rigid body simulations was an order of magnitude faster than their deformable counterparts. If cartilage stresses or strains are of interest to the user, a deformable cartilage representation would be necessary. However, this change to the finite element simulation is compatible with the segmentation and meshing workflow we have implemented. Similarly, our models did not include meniscal structures. Instead, we used the soft-tissue constraints of the tibiofemoral joint calibrated to match overall joint laxity measured in a cadaveric study—that is, researchers calibrated these ligament properties to compensate for the lack of a meniscus (Harris et al., 2016). Finally, we only examined contact mechanics of the tibiofemoral joint, which have historically been sensitive to geometry (Fitzpatrick et al., 2012, 2011; Navacchia et al., 2016; Gibbons et al., 2019). While this model provided us with numerical comparisons between the manual and automatic segmentations, additional experimental data is necessary to validate the resulting joint mechanics predictions. Additionally, further analysis is required to assess the sensitivity of joint kinematics, ligament mechanics, and joint loads to predicted labels and to quantify the effects of more sophisticated deep learning algorithms on FE simulation accuracy.

In combination with further advancements in deep learning, this framework represents a major advance in the study of natural knee biomechanics, and presents a feasible way to produce *population* sized finite element studies of the natural knee. The time required to produce quality hexahedral meshes has been reduced from a full workday of person-hours to 2 min. Additionally, we found that even the segmentation labels from our intermediate CNN models were useful during our bone segmentation process; manually correcting a percentage of suboptimal (DSC ∼65%) segmentation labels proved much faster than starting from scratch. Future researchers should not underestimate the time savings made possible by even a simple predictive model, and semi-supervised methods make it possible to train such models with limited data (Burton et al., 2020).

We’ve designed our *alpha-build* framework such that it may be adapted to any laminar structure approximated by a planar or cylindrical surface. Few modifications are necessary for other “hinge” joints, and the hip joint would only require the addition of a spherical coordinate raytracing function. The pipeline presented here has potential to improve our statistical shape and function models of the knee joint by better capturing population-based variation through inclusion of large-volume patient datasets. Integrating this pipeline with longitudinal patient datasets like the Osteoarthritis Initiative allows us to develop libraries of patient-specific models to quantitatively investigate relationships between anatomy, joint loading and longitudinal joint degeneration.

## Data Availability

The raw data supporting the conclusions of this article will be made available by the authors, without undue reservation.
